# Comparison of proximal contact tightness and contour of bioclear biofit HD versus Composi-tight 3DXR matrix systems in Class-II composite restoration: A randomized clinical study

**DOI:** 10.1016/j.jobcr.2025.04.005

**Published:** 2025-04-19

**Authors:** Shreya Volety, Ajay Singh Rao, Karkala Venkappa Kishan, Nimisha C. Shah, Dikshit Solanki, Geetanjali Jain

**Affiliations:** a Department of Conservative Dentistry and Endodontics, K.M Shah Dental College, Sumandeep Vidyapeeth, Piparia, Gujarat, Vadodara, India; b Department of Conservative Dentistry and Endodontics, Srinivas Dental College and Hospital, Mukka, Suratkal, Karnataka, Mangaluru, India

**Keywords:** Bioclear matrix system, Class-II restoration, Direct composite restoration, Garrison's matrix system, Proximal contact tightness, Proximal contour

## Abstract

**Objective:**

To assess proximal contact tightness and contour of two different sectional matrix systems in class-II direct composite restoration using newly designed assessment criteria.

**Materials and methods:**

In a double-blinded randomized clinical trial, 62 class-II direct composite restorations were performed in 62 patients using *Composi-Tight 3DXR* (n = 31) and *Bioclear Biofit HD* matrix systems (n = 31). Proximal contact tightness and contour were assessed using a self-designed criterion. Statistical analysis utilized *Chi-Square* and *Independent sample t-test*, with P < 0.001 considered significant.

**Results:**

The mean ± sd score for clinical and radiographical evaluation of proximal contact tightness of the *Bioclear Biofit HD* group (1.06 ± 0.25) was statistically significant with a p-value (<0.001) compared to the *Composi-Tight 3DXR* group (2.39 ± 0.72).

**Conclusion:**

The study found that the Bioclear Biofit HD matrix system demonstrated better proximal contact and contour than the *Composi-Tight 3DXR* Sectional Matrix System in Class-II direct composite restorations.

## Graphical abstract

**Figure:**
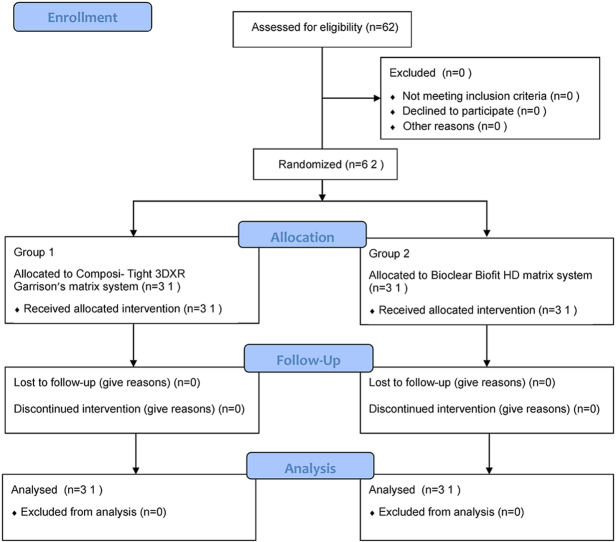

## 1 Introduction

Class-II composite restorations can be challenging due to the intricacies involved in properly contouring and adapting the composite material to the tooth's individual anatomy. Successfully rehabilitating a proximal surface while restoring a class-II cavity with a direct composite filling necessitates meeting various criteria. These criteria encompass achieving an anatomically correct contour and a tight contact area, ensuring proper marginal adaptation, and accurately placing the marginal ridge.^1^

Two key factors must be considered when recreating the proximal surface: contact tightness and surface contour. The strength of the contact is influenced by the size, position, and shape of the proximal contact areas (PCA), which are, in turn, determined by the proximal contours (PC) of the teeth in contact.^2^ Due to their thicker enamel than surrounding regions, the marginal ridges of the posterior tooth play a crucial role in enhancing the crown's structural integrity. Any loss of these ridges can significantly weaken the tooth.^3^

Over the past ten years, there has been a significant rise in demand for aesthetic restorations.^4^ Resin-based composite is currently one of the most extensively utilized materials in aesthetic dentistry.^4^^,^^5^ Composite resins' advantage is their strong bonding capability to enamel and dentin.^4^ However, achieving precise anatomic proximal contacts remains a challenge during the placement of direct posterior restorations. This issue arises from various factors, such as the inability to 'condense' composite material like amalgam, resulting in inadequate matrix adaptation to the neighboring tooth and polymerization shrinkage.^5^ Moreover, blood and tissue fluid contamination in Class-II composite restorations reduces bond strength, inhibits polymerization, and increases microleakage, emphasizing the need for proper isolation and decontamination.^6^

Research has focused on enhancing material characteristics and application methods to resolve present issues effectively. The matrix system and separation technique chosen are key considerations in this context. Although circumferential matrix systems have been commonly utilized, they have limitations when creating tight proximal contacts and achieving the desired proximal matrix form.^7^ Introduced in 1986, the sectional matrix system was substituted for circumferential bands. By separating rings, these bands effectively addressed the "push-pull" problem associated with circumferential bands, resulting in more dependable contacts even in cases with considerable space between teeth.^7^

The *Garrison Composi-Tight 3DXR* (GMS-3DXR) sectional matrix system is favored for its consistent results, but applying force can lead to distortions during placement, separation, stabilization, and material application.^8^

The *“bioclear Matrix System"* was introduced in 2007 and has gained significant popularity recently. This system uses transparent sectional matrix bands, enabling multi-angle light curing from the buccal, palatal, and occlusal sides, reducing polymerization shrinkage and microleakage.^1^ Its contoured design and curved occlusal embrasure improve adaptation and reduce finishing time.^4^ While the Bioclear matrix system has several benefits, its only drawback is that it demands precise technique, involves a learning curve, and may need additional time for proper adaptation and placement.^9^

The FDI World Dental Federation's evaluation standards for determining the tightness of proximal contacts are obsolete and insufficient in light of contemporary techniques and matrix systems.^10^^,^^11^ Henceforth, new self-designed criteria for Class-II composite restorations were established. The criteria were validated through expert input and statistical analysis and subsequently copyrighted by the Government of India.

While various studies have explored factors influencing proximal contact tightness (PCT), no direct comparison exists between *GMS-3DXR* and *Bioclear Biofit HD (BBHD)* sectional matrix systems.^2^^,^^4^^,^^5^ This study aims to fill this void by evaluating the reconstruction of proximal contact tightness and contour using these systems, employing the newly developed clinical and radiographic assessment criteria. The study's null hypothesis was that there would be no difference in proximal contact tightness and proximal contour between two sectional matrix systems in class-II direct composite restoration.

## 2 Materials and method

**Related approvals**: The research protocol and written informed consent were approved by the Institutional Ethics Committee (SVIEC/ON/DENT/BNPG21/D22051). Prior CTRI registration *(CTRI/2022/06/043116)* was done before commencing the study; The participants were chosen based on specific inclusion and exclusion criteria. The present study followed CONSORT guidelines (2010). All the treatment procedures were conducted according to the CDC COVID-19 Guidelines for dental settings.

**Sample size calculation**: Based on the previous article, a Chi-Square test for categorical data and the Independent sample *t*-test for quantitative data were employed. Considering a 1.00 mean difference in delta E between two groups with SD 0.93, a minimum of 52 samples (26 in each group) was taken with 99 % confidence and 90 % power.

**Inclusion criteria**: Patients between 18 and 60 years of age with unilateral Class-II Carious lesions in molars and premolars with an intact adjacent tooth were included in the study (GV Black's classification). Written informed consent was obtained from all patients, and only those willing to sign the consent form were included in the study.

**Exclusion criteria**: The study excluded patients with poor oral hygiene, severe periodontal disease, supraerupted teeth, heavy bruxism, diastema between posterior teeth, defects, and teeth that must be used as an abutment. Pregnant and lactating women were also excluded. Any history of lacerations, trauma, or inflammation on the external cheek was also excluded.

**Pre-operative assessment**: The carious teeth in all patients were subjected to clinical evaluation, and those carious lesions with a catch present while passing a probe in the proximal area and showing positive response on heat, cold test, and electric pulp testing were included. After that, the carious teeth in all the patients were subjected to radiographic evaluation using bite-wing radiographs by a paralleling technique using a long cone and X-ray positioned device for the confirmation of the extent of the lesion. Radiographs showing radiolucency involving enamel and dentin and not involving pulp were subjected to further clinical procedures.

**Randomization**: For randomization and allocation concealment, sixty-two numbers ranging from 1 to 62 were generated through computer software (http://www.random.org/). These numbers were then randomly assigned to either the intervention group (BBHD) or the control group (GMS-3DXR) using a Microsoft Excel sheet with a block randomization method consisting of 2 blocks, each with 31 patients. The allocation details were printed on a table, which was securely kept and accessed only by a co-investigator who was responsible for disclosing the assigned group of each patient upon contact.

GROUP 1 – Composi-Tight 3DXR Sectional Matrix System *(Garrison's Dental Solution)* (Control group) = 31.

GROUP 2- Bioclear Biofit HD matrix system (Experimental group) = 31.

**Clinical Procedure**: The clinical restorative procedure was started by performing the dental prophylaxis with a rubber cup in a contra-angled handpiece at low speed and pumice slurry to remove the residues on the tooth's surface. Quadrant Rubber dam isolation was done using a heavy gauge rubber dam sheet (*Nic Tone rubber dam, MDC Dental, Mexico*). The caries-detecting dye (*Kuraray Noritake Dental Inc., Pidilite Industries Limited, India*) was applied before caries removal. It allows the selective removal of only the affected tissue while preserving the healthy dentin. Caries were excavated using a sterile high-speed handpiece *(Nakanishi Inc, Kanda Toshima-cho, Chiyoda, Tokyo)* and using a round carbide bur *(Dentsply Sirona Inc, USA)*, an occlusal outline design and initial extension towards the proximal area is performed. A straight fissure diamond bur *(Mani Inc, Kiyohara Industrial Park, Utsunomiya, Tochigi, Japan)* was used to keep it parallel with the long axis of the tooth for occlusal convergence. Depending on the extent of caries, the proximal box was prepared to keep the facio-lingual width narrow and the axial wall with slight outward convexity. In a moderately deep carious lesion, the initial removal of caries was done using a carbide bur (*Dentsply Sirona, Inc, USA*) to access the compromised dentin structure. The infected dentin, which was soft and leathery in consistency, was removed with the aid of a dental spoon excavator *(Hu-Friedy Mfg. Co., LLC, Rockwell St. Chicago, USA)*, and the tooth preparation was limited to the removal of infected dentin, preserving the tooth structure. A periodontal probe was used to clinically measure the depth of Class II caries, accurately assessing the lesion's extent. If the cuspal width was <2 mm after caries excavation, the tooth was considered for cuspal coverage and excluded from the study. In cases where gingival overgrowth was present, it was removed by electrocautery or laser, and a bleeding control protocol was performed.

**Fig. 1:**
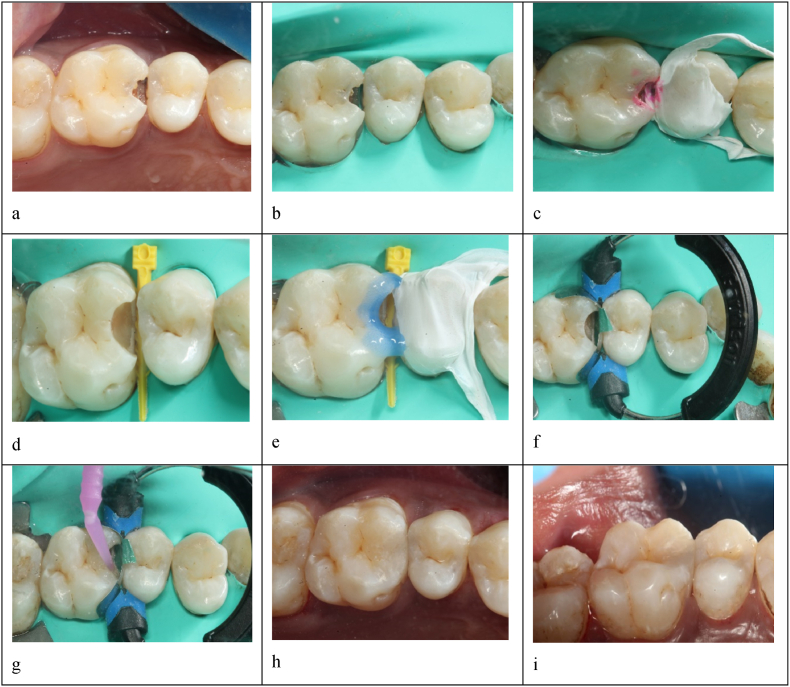
Class-II composite restoration using Composi- Tight 3DXR Garrison's Matrix system a – pre-operative clinical picture b – Rubber dam isolation c – Application of caries detecting dye d – Complete caries excavation e – Etching with 37 % phosphoric acid f – Composi- Tight 3DXR Garrison's Matrix system placement g - Bonding agent application h – post-operative occlusal view of Class-II restoration i - Palatal view for proximal contact.

**Fig. 2:**
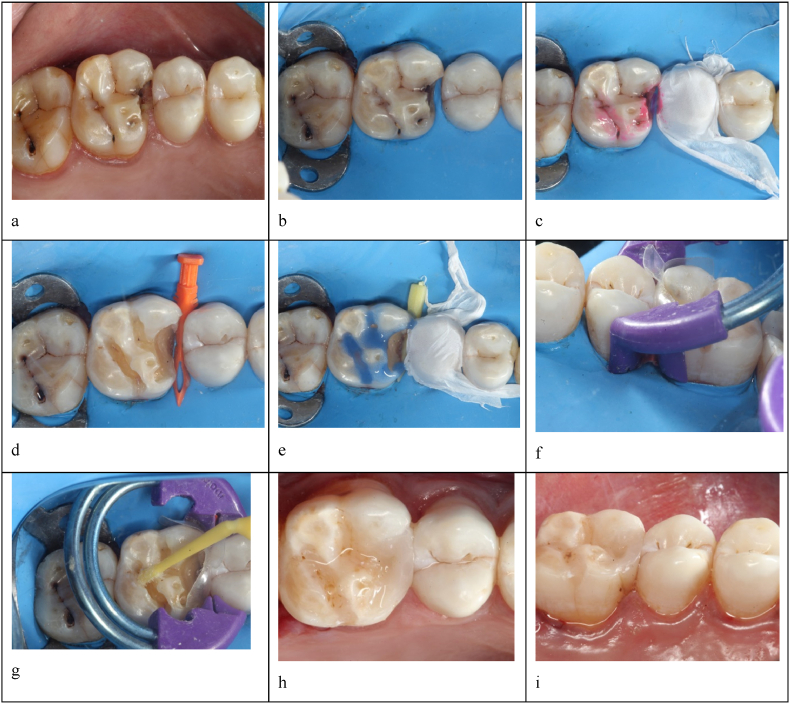
Class-II composite restoration using Bioclear Biofit HD matrix system.

**Fig. 3:**
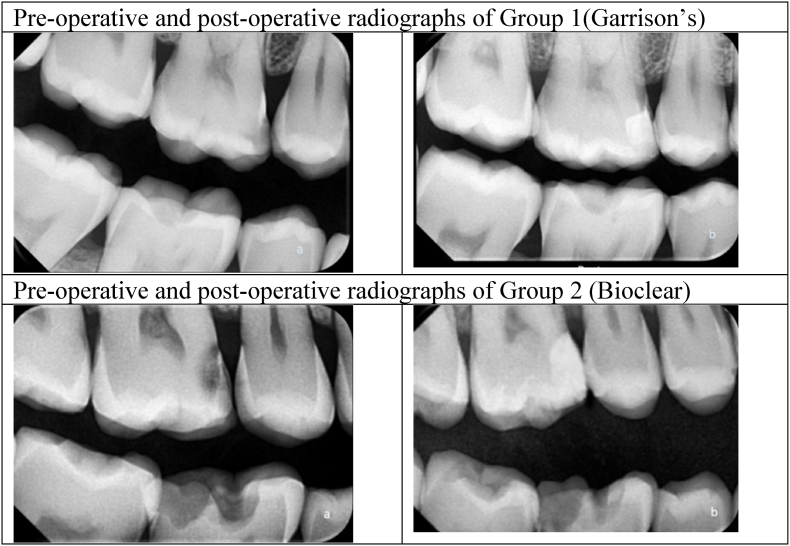
Pre-operative and post-operative radiographs of both groups.

At first, a pre-operative clinical picture and bite-wing radiograph were taken >Fig. 1, Fig. 2, Fig. 3. Later, shade selection was done before using the composite button technique. Quadrant Rubber dam isolation was done using a heavy gauge rubber dam sheet (*Nic Tone rubber dam, MDC dental, Mexico*)Fig. 1, Fig. 2. The caries-detecting dye *(Kuraray Noritake Dental Inc., Pidilite Industries Limited, India)* was applied before caries removal Fig. 1, Fig. 2. After the complete removal of dental caries [Fig Id, IId], selective enamel etching with 37 % phosphoric acid followed by universal bonding agent application to the enamel and dentinal walls was done Fig. 1, Fig. 2. This was followed by the proximal wall build-up and incremental cusp by cusp addition of nano-hybrid resin. Each increment was light-cured *(Orikam Curing Pen Light Cure Unit, Orikam Healthcare India Private Limited, Haryana, India)* for approximately 20 s, as recommended by the manufacturer, to ensure complete and effective polymerization. After completing the composite placement, the standard finishing and polishing regime was carried out. A final post-operative clinical picture and a bite-wing radiograph were taken Fig. 1, Fig. 2, Fig. 3.

In group 1 (control group), *the GMS-3DXR* (*Garrison's Dental Solution, Michigan, USA*) matrix system was used Fig. 1f,g for proximal wall build-up. First, the selected contoured sectional matrix band that most closely approximates the occluso-gingival height of the tooth was placed, and the wedge was inserted using Garrison's Wedge Wands or another Garrison wedge by keeping one finger on the matrix band to prevent the wedge from dislodging the band during insertion. The 3DXR-ring retainer was placed with the Composi-Tight Ring Placement forceps over the wedge. The matrix band was firmly pushed into contact with the adjacent tooth in the desired contact area, and the Class-II composite restoration was carried out by incremental cusp-by-cusp addition of nano-hybrid resin composite. After the composite placement was completed, the standard finishing and polishing regime was carried out.

In group 2 (experimental group), the BBHD (*Bioclear Matrix Systems, Washington*) matrix system was used Fig. 2f,g for proximal wall build-up. First, the tab of the selected matrix was held with a tweezer, and the matrix band was slid into the embrasure space such that it was centered on the tooth and the tab was facing occlusal. The selected bioclear diamond wedge was inserted to ensure that it did not invert the curve of the bioclear matrix. Then, the Twin ring was placed using bioclear ring forceps just enough to get past the matrix down to the gingival margin, and restoration was carried out by incremental cusp-by-cusp addition of nano-hybrid resin composite. After the composite placement was completed, the standard finishing and polishing regime was carried out.

**Clinical and Radiographic evaluation:** Before implementation, a prior statistical validation test, namely the *Cronbach alpha test*, was carried out to ensure the reliability of the criteria. Experts conducted content validation, yielding a statistically significant mean from the validation.

Consequently, the class-II restoration's clinical and radiographic proximal contact tightness was evaluated based on the copyrighted self-designed criteria (Table I). These criteria assessed PCT for Class-II composite restorations in patients clinically utilizing dental floss.

**Table 1:** Clinical and radiographic evaluation of proximal contact tightness and proximal contour in Class-II composite restoration.

CLINICAL EVALUATION			
SR.NO	CRITERIA	SCORE	DESCRIPTION
1.	Proximal contact tightness	1	Normal contact (clinically floss or 25 μm metal strip can pass)
		2	Tight contact (clinically floss or 25 μm metal strip can only pass with pressure)
		3	Clinically Acceptable (floss can pass easily and metal strip of 50 μm can pass)
		4	No contact (clinically unsatisfactory with possible damage due to food impaction and metal strip of 100 μm can pass easily)
2.	Proximal contour	1	Normal contour (adequate convexity)
		2	Over contoured (convexity lightly compromised, repair is possible)
		3	Under contoured (convexity severely compromised, re-restoration is required)

RADIOGRAPHIC EVALUATION			
SR.NO	CRITERIA	SCORE	DESCRIPTION
1.	Proximal contour and contact	1	Adequate contour and contact.
		2	Acceptable contour and slight contact.
		3	Compromised contour and contact
		4	No contour and open contact.
2.	Marginal adaptation	1 2 3 4	Adequate Acceptable (with a thin radiolucent adhesive resin line/cement observed at the margin) Compromised (Radiographically visible radiopaque overhang) Not acceptable (Underfilled with radiolucency at gingival seat margin)

SCORE	INFERENCE
4–7	Excellent restoration
8–11	Acceptable restoration (with minor corrections)
11–15	Not acceptable restoration (Re-restoration is required)

The present study followed the CONSORT 2010 guidelines. The statistical analysis was performed using SPSS software version 22.

**Results:** Statistical analysis was performed using the *Chi-Square test* for categorical data along with the *Independent sample t-test* for quantitative data. Table II depicts the demographic data of groups 1 and 2. There were 15 males and 16 females in both group 1 and group 2, resulting in a total of 31 patients in both group 1 and group 2. The age range was from 18 to 60 years. The study included 48 molars and 14 premolars with class-II carious lesions, of which 28 were mesio-occlusal caries and 34 were disto-occlusal caries. On comparison, the mean ± sd score for clinical evaluation of the proximal contour of group 2 (1.0 ± 0.0) is better than that of group 1 (1.74 ± 0.58) and is statistically significant with a p-value (<0.001) (Table III). On comparison, the mean ± sd score for radiographic evaluation of proximal contact and contour of group 2 (1.1 ± 0.3) is better than that of group 1 (2.06 ± 0.51). It is statistically significant with a p-value (<0.001) (Table III). On comparison, the mean ± sd score for radiographic evaluation of cervical adaptation of group 2 (1.0 ± 0.0) is better than that of group 1 (1.71 ± 0.78) and is statistically significant with p-value (<0.001) (Table III).

**Table 2:** Demographic data.

			Group	
			Group 1	Group 2
Gender	Female	Count	16	16
		% within Group	48.4 %	48.4 %
	Male	Count	15	15
		% within Group	51.6 %	51.6 %
Total		Count	31	31
		% within Group	100.0 %	100.0 %

**Table 3:** Comparison of clinical and radiographic proximal contact tightness, proximal contour, and radiographic cervical adaptation between Group 1 and Group 2.

	Group 1(n = 31)	Group 2(n = 31)	t	P VALUE
	Mean ± sd	Mean ± sd		
Clinical evaluation proximal contact tightness	2.39 ± 0.72	1.06 ± 0.25	9.718	**<0.001**
Clinical evaluation proximal contour	1.74 ± 0.58	1 ± 0	7.178	**<0.001**
Radiographic evaluation proximal contact and contour	2.06 ± 0.51	1.1 ± 0.3	9.073	**<0.001**
Radiographic evaluation cervical adaptation	1.71 ± 0.78	1 ± 0	5.047	**<0.001**
Total score	7.9 ± 1.47	4.1 ± 0.3	14.137	**<0.001**

## 3 Discussion

Restoration of interproximal carious lesions poses a distinct challenge for dental professionals due to various factors: Interproximal carious lesions form between neighboring teeth, making them hard to reach with conventional dental tools.^12^ One research highlighted that disto-occlusal caries were the most prevalent type of class-II caries, particularly among females aged 25–35 in the first quadrant.^13^ The ideal PCA is a barrier against food impaction, contributing to underlying periodontal health. It provides a food spillway and facilitates hygienic cleaning.^14^

According to a study, for optimal proximal geometry, it is important that the PC of a class-II composite restoration closely resembles that of an intact natural tooth. In the natural dentition, it is suggested that the interproximal contact area should measure approximately 1.5–2 mm rather than being a mere point.^1^ Once innovative, G.V. Black's cavity designs are acknowledged for their limitations, especially in posterior teeth, where sharp internal angles and the connection between occlusal and interproximal areas may cause restoration failure. Fractures often begin at dentin line angles. Interrupted cavities show better crack resistance than connected ones. The rise of tooth-colored restorative materials, like resin-based composites, has shifted towards more minimally invasive cavity preparations.^15^

It should be remembered that the results of the current study's findings rely on the possible reasons that the BBHD matrix bands allowed for better adaptation to the tooth surface and ensured optimal contouring of the restoration, promoting excellent embrasure anatomy. Using transparent matrices in the bioclear system enhanced light transmission during light-curing. This allegedly resulted in more effective polymerization of the underlying resin composite, ensuring thorough curing and reducing the risk of incomplete polymerization.^4^^,^^16^

For resin-based composites, features like parallel walls and resistance and retention form, which are beneficial for amalgam, may hinder outcomes. Polymerization shrinkage, inherent to composites, can be mitigated with adapted cavity preparation techniques. Clinicians can reduce restoration failure risk and enhance posterior composite outcomes by accommodating composite properties.^15^^,^^16^

A study conducted on caries-detecting dye stated that using caries-detecting dyes is particularly advantageous in minimizing the excessive removal of carious dentin. It allows selective removal of only the affected tissue while preserving the healthy dentin.^17^ This conservative method helps maintain the tooth's structural integrity and reduces the risk of pulp exposure or unnecessary loss of tooth structure.^18^

Two main factors are crucial in creating contact: surface contour and tooth separation. Traditional straight metal matrix bands, used for amalgam restorations, pose limitations in class-II composite cases. They often result in narrow occlusal-gingival contacts, lacking stability and support, leading to issues like food impaction and periodontal problems.^1^^,^^14^ Straight bands also yield thinner marginal ridges, are less fracture-resistant, and are prone to chipping under occlusal forces, compromising restoration durability. Moreover, they create inferior proximal contact with decreased tightness, emphasizing the need for contoured matrix bands in composite restorations.^1^^,^^14^^,^^19^^,^^20^ As a result, sectional matrix bands were introduced to improve the accuracy and excellence of dental restorations.^4^ These sectional matrix systems consist of flexible and contoured metal or plastic bands that can be effortlessly adjusted to fit the unique anatomy of every tooth, thereby overcoming the shortcomings of traditional circumferential matrix bands.^21^ According to a study, the sectional band with a separation ring was associated with stronger contact points than the circumferential band.^22^^,^^23^

The shift to sectional matrices for class-II composite restorations has led to a notable improvement in proximal contour and contact outcomes as opposed to traditional circumferential systems. Using contemporary pre-contoured sectional matrices alongside separation rings enhances adaptation to the tooth surface.^22^ Different separation rings feature tines in parallel, divergent, or V-shaped arrangements, influencing the matrix band's ability to conform to the tooth's anatomy.^24^ The GMS-3DXR was believed to provide predictable proximal contact among the ring systems currently available.^8^ Hence, in this study, *GMS-3DXR* was used as a control group among the available pre-contoured metal matrix systems for class-II restoration. However, in the study conducted by *Loomans* et al., the V-ring of the *Palodent V3 sectional matrix system (Dentsply)* showed the least amount of proximal overhang when compared with the *Composi-Tight Gold* matrix system. This difference may be due to the V-configuration of the tines in the buccal-lingual direction, resulting in a more precise adaptation of the matrix to the tooth.^24^ Moreover, while using *GMS-3DXR*, burnish or secure matrices adjacent to the neighboring tooth are suggested to ensure proper embrasure anatomy and facilitate thorough cleaning of the contact area. This necessitates additional techniques to aid the operator in applying pressure on the contact region during light curing *(InstruDent Woodpecker LED D Dental Curing Light Unit, India).*^4^^,^^15^

The bioclear matrix system (BBHD) addresses the need for adequate embrasure anatomy and facilitates proper contact cleaning by offering unique features that overcome common challenges encountered with traditional matrix systems. Compared to other matrix systems, these bands offer several advantages over the pre-contoured metal sectional matrix system. Based on a few studies, the transparent sectional matrix bands mimic the natural contour of the tooth. This allows for better adaptation to the proximal surface, forming tight and stable proximal contacts. This allows for better adaptation to the tooth surface and ensures optimal contouring of the restoration, promoting excellent embrasure anatomy.^1^^,^^15^ An in-vivo study compared the *Palodent V3 sectional matrix system (Dentsply)* with the BBHD (*Biofit HD Posterior (Bioclear)*.^4^ They concluded that utilizing a transparent matrix with the injection molding technique significantly reduced post-operative sensitivity. The study showed that the *BBHD* matrix system performed better in proximal contact tightness than the *GMS-3DXR* matrix system. A systematic review showed that class-II posterior composite resin restorations placed with a combination of sectional matrices and separation rings resulted in a stronger proximal contact than when a circumferential matrix system was used, similar to the present study.^25^

Based on the limitations of the analysis and the assumption made, a statistically significant difference was seens in the clinical and radiographic proximal contact tightness between the experimental group *(Bioclear Biofit HD matrix system)* and the control group *(Composi Tight 3DXR, Garrison's matrix system)*. Hence, the null hypothesis for this study was rejected.

## 4 Strength

This study's strength lies in its innovative approach to evaluating the tightness of proximal contacts and the contour of the matrix system. By utilizing a newly developed scoring system, this research not only enhances the accuracy of assessing class-II restoration's contact and contour but also provides a more detailed understanding of different matrix systems' effectiveness and precision. This unique method ensures more reliable and comprehensive results, significantly advancing dental restoration techniques.

## 5 Limitation

The study's main limitation was its small sample size. Further clinical studies are essential to compare various matrix systems with larger samples. Additionally, investigating the time required for restoration using specific matrix systems is crucial for comprehensive evaluation. These efforts will provide a more robust understanding of the efficacy and efficiency of different techniques in dental restorations.

## 6 Conclusion

Within the study's limitations, it was concluded that the class-II composite restorations using *the Bioclear Biofit HD matrix system* performed better, with significant optimum proximal contact and no overhanging margins, compared to the *Composi Tight 3DXR (Garrison's matrix system).*

## Contribution details (to be ticked marked as applicable)

Shreya Volety: Literature search, Data acquisition, Data analysis, Manuscript preparation, Manuscript review, Guarantor. Ajay Singh Rao: Concepts, Design, Definition of intellectual content, Data analysis, Manuscript editing, Data acquisition, Manuscript review. Karkala Venkappa Kishan: Concepts, Design, Manuscript editing, Manuscript review. Nimisha C Shah: Concepts, Design, Manuscript editing, Manuscript review. Dikshit Solanki: Concepts, Design, Manuscript editing, Manuscript review. Geetanjali Jain: Concepts, Design, Manuscript editing, Manuscript review.

## Funding

THIS IS A SELF-FUNDED STUDY WITHOUT 10.13039/100026479ANY EXTERNAL FUNDING DONE FOR 10.13039/501100000725THE SAME.

## Declaration of competing interest

I am writing to formally submit my manuscript titled “**Comparison of Proximal Contact Tightness and Contour of Bioclear Biofit HD versus Composi-Tight 3DXR Matrix Systems in Class-II Composite Restoration: A Randomized Clinical Study”** for consideration for publication in the *Journal of Oral Biology and Craniofacial Research*. This manuscript represents original research conducted by my co-authors and me, and we believe it contributes valuable insights to the field of oral biology and craniofacial research.

I hereby declare that there are no conflicts of interest associated with this manuscript. All authors have contributed significantly to the work, reviewed the final manuscript, and approved its submission to your esteemed journal. Additionally, this manuscript has not been published elsewhere, nor is it under consideration for publication in any other journal.

We confirm that ethical guidelines have been followed, and any necessary institutional approvals have been obtained. Any funding sources or financial support for this research have been disclosed within the manuscript.

We sincerely appreciate your time and consideration, and we look forward to the opportunity to contribute to the journal. Please do not hesitate to contact me should you require any further information.

## References

[1] Peumans M., Venuti P., Politano G., Meerbeek B.V. (2021). Effective protocol for daily high-quality direct posterior composite restorations. The interdental anatomy of the class-2 composite restoration. J Adhesive Dent.

[2] Kumari S., Raghu R., Shetty A., Rajasekhara S., Padmini S. (2023). Morphological assessment of the surface profile, mesiodistal diameter, and contact tightness of Class II composite restorations using three matrix systems: an in vitro study. J Conserv Dent.

[3] Shivakumar A., Kalgeri S., Dhir S. (2015). Clinical considerations in restorative dentistry - a narrative review. J Int Clin Dent Res Organ..

[4] Rao D.M., Narayana V., Keshava Prasad B.S. (2022). Sectioning through sectional matricing techniques: an in-vivo comparative evaluation of post-operative sensitivity. J Med Dent Sci.

[5] Wirsching E., Loomans B.A.C., Klaiber B., Dörfer C.E. (2011). Influence of matrix systems on proximal contact tightness of 2- and 3-surface posterior composite restorations in vivo. J Dent.

[6] Aggrawal V., Logani A., Jain V., Shah N. (2008). Effect of cyclic loading on marginal adaptation and bond strength in direct vs. indirect class II MO composite restorations. Operat Dent.

[7] Kannan K., Hima A.S. (2022). Matrix system preferred for class II composite restoration in university setup. J Res Med Dent Sci.

[8] The Use of Separating Rings in the Placement of Class-II Composite-Resins. Available at: https://www.garrisondental.com/case-study/use-separating-rings-placement-class-ii-composite-resins.

[9] El-Shamy H. (2018). Influence of metal versus transparent matrices on proximal contact tightness of Class II bulk-fill composite restorations. Egypt Dent J.

[10] Hickel R., Peschke A., Tyas M. (2010). FDI World Dental Federation: clinical criteria for the evaluation of direct and indirect restorations—update and clinical examples. Clin Oral Invest.

[11] Marquillier T., Doméjean S., Le Clerc J. (2018). The use of FDI criteria in clinical trials on direct dental restorations: a scoping review. J Dent.

[12] Covey D., Schulein T.M., Kohout F.J. (1989). Marginal ridge strength of restored teeth with modified Class II cavity preparations. J Am Dent Assoc.

[13] R K., Nasim I., Chaudhary M. (2020). Prevalence of proximal caries in the posterior teeth in patients visiting a dental college. J Complement Med Res.

[14] Chuang S.F., Su K.C., Wang C.H., Chang C.H. (2011). Morphological analysis of proximal contacts in class II direct restorations with 3D image reconstruction. J Dent.

[15] Clark D. (2007; 26(10):122,124,126-7). Introducing the clark class II restoration. Dent Today.

[16] Clark D. (2013). The new science of strong teeth: class II preps. https://pubmed.ncbi.nlm.nih.gov/23802379/.

[17] Sano H. (1987). Relationship between caries detector staining and structural characteristics of carious dentin. J Stomatol Soc Jpn.

[18] Oikawa M., Itoh K., Kusunoki M., Kitahara N., Miyazaki T. (2013). Efficacy of three caries-staining agents. DMR, dental. Med Reserve.

[19] Loomans B.A.C., Roeters F.J.M., Opdam N.J.M., Kuijs R.H. (2008). The effect of proximal contour on marginal ridge fracture of Class II composite resin restorations. J Dent.

[20] Peumans M., Van Meerbeek B., Asscherickx K. (2001). Do condensable composites help to achieve better proximal contacts?. Dent Mater.

[21] Bailey O. (2021). Sectional matrix solutions: the distorted truth. Br Dent J.

[22] Loomans B.A.C., Opdam N.J.M., Roeters F.J.M., Bronkhorst E.M., Burgersdijk R.C.W., Dörfer C.E. (2006). A randomized clinical trial on proximal contacts of posterior composites. J Dent.

[23] Asif M., Khattak I., Qureshi A., Zain M., Aslam N., Khan M.I. (2023). Comparison between two types of matrix systems for contact tightness in class-II composite restorations. J Ayub Med Coll Abbottabad.

[24] Loomans B.A.C., Opdam N.J., Roeters F.J.M., Huysmans M.C.D. (2012). Proximal marginal overhang of composite restorations in relation to placement technique of separation rings. Operat Dent.

[25] Anantula K., Vankayala B., Yadav S.S. (2024). Proximal contact tightness of direct Class II composite resin restorations with various matrix systems: a systematic review. J Conserv Dent.

